# α,α-disubstituted β-amino amides eliminate *Staphylococcus aureus* biofilms by membrane disruption and biomass removal

**DOI:** 10.1016/j.bioflm.2023.100151

**Published:** 2023-08-25

**Authors:** Dominik Ausbacher, Lindsey A. Miller, Darla M. Goeres, Philip S. Stewart, Morten B. Strøm, Adyary Fallarero

**Affiliations:** a Natural Products and Medicinal Chemistry Research Group, Department of Pharmacy, UiT The Arctic University of Norway, N-9037, Tromsø, Norway; b Center for Biofilm Engineering, Montana State University, Bozeman, MT, 59717, USA; c Drug Research Program, Division of Pharmaceutical Biosciences, Faculty of Pharmacy, University of Helsinki, FI-00014, Helsinki, Finland

## Abstract

Bacterial biofilms account for up to 80% of all infections and complicate successful therapies due to their intrinsic tolerance to antibiotics. Biofilms also cause serious problems in the industrial sectors, for instance due to the deterioration of metals or microbial contamination of products. Efforts are put in finding novel strategies in both avoiding and fighting biofilms. Biofilm control is achieved by killing and/or removing biofilm or preventing transition to the biofilm lifestyle. Previous research reported on the anti-biofilm potency of α,α-disubstituted β-amino amides **A1**, **A2** and **A3**, which are small antimicrobial peptidomimetics with a molecular weight below 500 Da. In the current study it was investigated if these derivatives cause a fast disintegration of biofilm bacteria and removal of *Staphylococcus aureus* biofilms. One hour incubation of biofilms with all three derivatives resulted in reduced metabolic activity and membrane permeabilization in *S. aureus* (ATCC 25923) biofilms. Bactericidal properties of these derivatives were attributed to a direct effect on membranes of biofilm bacteria. The green fluorescence protein expressing *Staphylococcus aureus* strain AH2547 was cultivated in a CDC biofilm reactor and utilized for disinfectant efficacy testing of **A3**, following the single tube method (*American Society for Testing and Materials designation number E2871)*. **A3** at a concentration of 90 μM acted as fast as 100 μM chlorhexidine and was equally effective. Confocal laser scanning microscopy studies showed that chlorhexidine treatment lead to fluorescence fading indicating membrane permeabilization but did not cause biomass removal. In contrast, **A3** treatment caused a simultaneous biofilm fluorescence loss and biomass removal. These dual anti-biofilm properties make α,α-disubstituted β-amino amides promising scaffolds in finding new control strategies against recalcitrant biofilms.

## 1 Introduction

The lack of innovative antibiotics poses a serious threat to human health [1]. Antimicrobial resistance leads to more than 35 000 deaths in the EU each year, according to estimates presented in a recently released report [2]. Not just resistant bacteria, but also bacterial biofilms with an intrinsic tolerance to antibiotics complicate successful treatment of infections [3]. On the other hand, industrial installations in sectors such as oil and gas, water supply and food processing suffer of biocorrosion and contamination due to biofilms. Countermeasures are costly and require expenses of many hundred billions of dollars [4]. Biofilms develop when planktonic bacteria form agglomerates that can adhere to a surface [5]. In patients, catheters, artificial heart valves or prosthetic joints are prone to bacterial attachment and biofilm formation [6]. In addition, biofilms can also be the cause of faulty wound healing and wound chronicity [7]. Biofouling on shipping vessels or leaks in oil and gas pipelines due to microbially influenced corrosion cause not just undesired investments but have a heavy environmental impact, too [4]. Besides bacteria, these agglomerates consist of an extracellular biofilm matrix, which contributes to the formation of three-dimensional biofilm structures and microenvironments [8]. Specialized bacteria exist in a biofilm, such as slow metabolizing persisters, or bacteria with high mutation rates, that promote environmental adaption of biofilms and high tolerance against antimicrobial treatments [8,9]. These features make biofilms a challenging target for treatment, especially in health care settings where harsh physical and chemical treatments are not an option. New approaches, including effective anti-biofilm compounds, are needed in order to develop new strategies against these recalcitrant pathogen formations [10]. Biofilms can be controlled by killing and/or removing the biofilm or preventing transition to the biofilm lifestyle [11,12] High-throughput methods are commonly used for screening of libraries consisting of natural products or synthetic molecules in order to identify compounds with anti-biofilm properties [13,14]. Besides identifying potential hits and development of lead compounds, substantial efforts have been invested in determining the mode-of-action of new compounds [15,16]. These studies are crucial for improvements regarding potency and toxicity, drug formulation, and administration, but also regarding utilizing synergism and avoiding antagonism in combination treatments [17,18]. In the medical community, anti-biofilm compounds approved by the regulatory agencies do not exist and the only chance to fight microbial biofilms is by using antibiotics or surgical removal of biofilm infected tissues or replacement of medical devices. One of most recent and innovative antibiotics introduced to the market is the antimicrobial depsipeptide daptomycin. This drug was approved by the Food and Drug Administration back in 2003 but innovative antimicrobial drugs or compound classes with a clearly novel mechanism of action are needed now and will be needed in the future [19]. A promising compound is the natural product teixobactin. This antimicrobial peptide is in late-stage preclinical development and potent against multi-resistant gram-positive bacteria [20,21]. Both, daptomycin and teixobactin have rather complex structures with potential attack points for degradation. Hydrolases in actinomycete WAC4713, for instance, have been reported to confer resistance by being able to hydrolyze the depsipeptide ester-bond in daptomycin [22]. Of note, the same structural element is present in teixobactin, however, no resistance development could be observed *in vitro*, yet [20,21]. Therefore, many groups investigate how to utilize the mode of action of natural products and/or potent antimicrobial peptides while equipping them with drug-like properties [23]. The group of Strøm has shown that it is possible to create novel and potent compounds by simplifying and condensing complex structures of natural products [[24], [25], [26]]. Hansen et al. for instance were able to transfer the antimicrobial motif of larger antimicrobial peptides to small molecules with enzymatic stability [27]. Furthermore, the group developed so called α,α-disubstituted β-amino amides which are peptidomimetics showing potency in the same range as larger antimicrobial and anticancer peptides [28,29]. These derivatives are easily synthesized, have a preference for gram-positive bacteria, including antibiotic resistant strains like MRSA and MRSE and have drug-like properties [28,30]. The activity spectrum also includes *Staphylococcus aureus* (*S. aureus*) biofilms, and both *in vitro* and microscopy studies have suggested that these derivatives possess microbicidal and biofilm removal properties [31]. In the present study, we investigated if these derivatives act on the bacterial membrane in *S. aureus* biofilms. This suggests that membrane related effects, like for instance permeabilization, would occur relatively fast. Quantitative and qualitative assays on biofilms formed in 96-well plates were conducted similar to a previously used screening setup, however using much shorter incubation times [31]. The control compounds chlorhexidine (CHX) and cetylpyridnium bromide (CTAB) have been included for comparison. Both compounds have similarities with the α,α-disubstituted β-amino amides, like cationic charge and an amphipathic structure. In addition, CHX and CTAB have a membrane perturbing mechanism of action [32]. CHX and **A3,** were further challenged with *S. aureus* biofilms that were cultivated in a CDC biofilm reactor. Disinfectant efficacy testing by using an adaption of *American Society for Testing and Materials* (ASTM) standard method E2871 [33] guided the confocal laser scanning microscopy studies using a treatment flow cell. A reporter system consisting of green fluorescent protein (GFP) expressing biofilm bacteria together with calcein red-orange fluorescence staining was used in order to determine if, and to which extent, bacterial membranes, and the biofilm as a whole, were affected during treatment with **A3**.

This knowledge will help to tune derivative potency in future and to further explore derivative properties in suitable formulations and applications for tackling biofilm challenges in health care and/or industrial settings.

## 2 Material and methods

### 2.1 The α,α-disubstituted β-amino amides A1 - A3 and chemicals

**Fig. 1:**
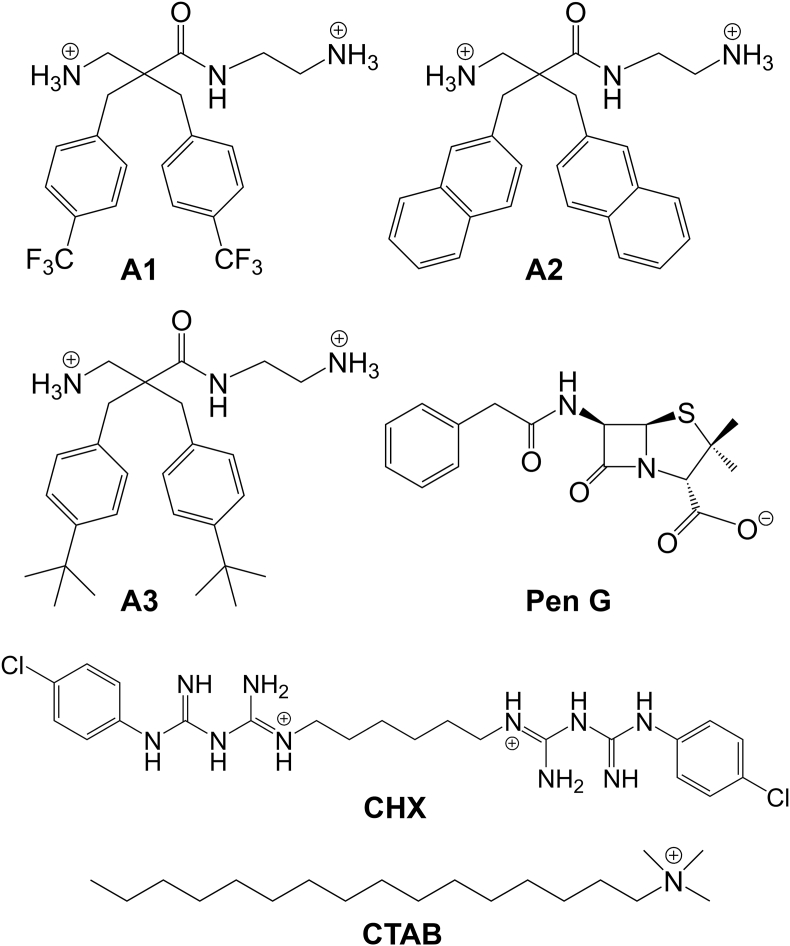
Compounds with antimicrobial properties used for mechanistic investigations on *S. aureus* biofilms. Representation of amphipathic α,α-disubstituted β-amino amides lead structures **A1** - **A3** with anti-biofilm properties and the disinfectants chlorhexidine (CHX) and cetylpyridnium bromide (CTAB) in cationic state. Penicillin G was used as control antibiotic represented as anion.

The α,α-disubstituted β-amino amides (**A1** - **A3,** Fig. 1) were synthesized according to a previously published procedure [28]. The derivatives were isolated as di-trifluoroacetate salts and purity above 95% was determined with an analytical RP-HPLC C_18_-column and UV detection at 214 and 254 nm. Prior to the experiments stock solutions of all derivatives were prepared in DMSO. DMSO concentrations did not exceed 2% and were well tolerated by *S. aureus* biofilms as reported earlier [34]. Chlorhexidine dichloride (CHX), cetyltrimethylammonium bromide (CTAB) and penicillin G sodium (Pen G) (all Sigma Aldrich, Schnelldorf, Germany) were used as treatment controls (Fig. 1). Autoclaved Milli-Q water was used to prepare stock solutions of CTAB and Pen G whereas DMSO was used for CHX.

### 2.2 Bacterial strains and growth condition

The reference strain *S. aureus* ATCC 25923 and the green fluorescent protein (GFP) expressing *S. aureus* strain AH2547, kindly provided by Dr. Alex Horswill, were used for the experiments. AH2547 contains the plasmid pCM29 [35]. For AH2547 tryptic soy broth (TSB) and tryptic soy agar (TSA) were supplemented with 10 μg/ml chloramphenicol for plasmid retention.

All strains were stored as glycerol stocks at −70 °C and TSA streak plates were prepared prior to the experiments. Three colonies were picked, inoculated in TSB and incubated at 37 °C and 200 rpm, overnight. For well-plate based assays the liquid cultures were prepared by diluting the overnight cultures 1000 times in fresh TSB. The inoculum was incubated under aerobic conditions at 37 °C, 250 rpm until late exponential growth was reached (4 h), corresponding to a bacterial concentration of 10^8^ CFU/ml. Biofilms were formed by transferring 200 μl of exponentially grown suspensions (10^6^ CFU/ml) to 96-well plates (Nunclon™ Δ surface; Thermo Fisher Scientific, Vantaa, Finland). Subsequently, the cultures were incubated for 18 h under equivalent conditions as stated above and described earlier [34].

For experiments involving the CDC biofilm reactor, biofilms were formed on glass coupons according to ASTM Method E2562 [36] and Buckingham-Meyer et al. [37]. A CDC reactor containing 500 ml full strength TSB and chloramphenicol (10 μg/ml) was inoculated with 1 ml of a 10^9^ CFU/ml overnight GFP expressing *S. aureus*. The biofilms were grown in batch conditions at 37 °C, 125 rpm for 24 h. Subsequently, continuous flow with one-tenth TSB was applied for another 24 h at 37 °C and 125 rpm until start of sampling.

### 2.3 Assessment of metabolic activity and biomass after 1 h treatment of *S. aureus* ATCC 25329 biofilms

The impact of α,α-disubstituted β-amino amides on biofilms of *S. aureus* ATCC 25923 formed in 96-well plates was investigated by two different staining methods as described earlier [31]. To investigate the immediate response of biofilms after exposure to the derivatives, biofilms were formed for 18 h, as described above. The planktonic phase was replaced with fresh TSB or compound containing TSB. Based on previous studies, derivatives at concentrations of 2xIC_50_ (50 μM for **A1** and **A2**; 45 μM for **A3**) and 50 μM of CHX and CTAB as well as 400 μM of Pen G were added. Untreated controls were treated with an equivalent of DMSO in TSB and the plates were incubated for 1 h. At the end of the exposure periods, the planktonic phase was carefully replaced with 20 μM resazurin in PBS (Lonza Walkersville Inc., Walkersville, USA) and the 96-well plate was incubated (200 rpm, RT, 40 min, darkness). Fluorescence was measured (λex 570 nm, λem 590 nm) with a Varioskan Multimode reader (Thermo Fisher Scientific, Vantaa, Finland). Metabolic activity was determined as percentage of untreated control. Subsequently, the supernatant was gently removed and 170 μl of the crystal violet solution was added and incubated for 5 min. The dye was removed and wells washed twice with deionized water. The dye was dissolved in 96% ethanol (200 μl/well). After 1 h the photometric absorbance (*λ* 590 nm) was measured using a Varioskan Multimode reader. Susceptibility of the *S. aureus* strain AH2547 to our derivatives was determined by using a similar 96-well setup. GFP production of the strain was exploited and loss of GFP fluorescence was recorded after exposure to biofilm treatments, further described in the supplementary information to this article.

### 2.4 Qualitative and quantitative detection of membrane integrity by SYTO 9 and propidium iodide staining

A membrane integrity assay and fluorescence microscopy studies (LIVE/DEAD BacLight Bacterial Viability Kit; EVOS FL imaging system, Thermo Fisher Scientific, Vantaa, Finland) were performed according to the manufacturer's instructions. Formed biofilms were exposed to treatments as described above for 1 h and staining was subsequently applied. Fluorescence of green SYTO 9 and red propidium iodide (PI) was determined using a Varioskan Multimode reader and the EVOS FL imaging system was used for fluorescence microscopy.

### 2.5 Detection of intracellular ATP leakage from *S. aureus* ATCC 25329 biofilms

The ATP leakage assay was adapted from Manner et al. [38]. In brief, *S. aureus* ATCC 25923 biofilms were formed and treated as described above. Untreated biofilms and negative control wells were incubated with TSB. After 1 h, the planktonic suspensions were removed and collected for each treatment. The suspensions were filtered using 0.22 μm syringe filters. Subsequently, 4 × 100 μL of each filtrate were transferred to a clear-bottom 96-well plate (Isoplate-TC; PerkinElmer, Waltham, MA, US). The CellTiter-Glo® reagent (Promega, Madison, WI, US) was prepared according to the manufacturer instructions. Luciferin luminescence was measured using Varioskan Flash Multimode Plate Reader.

### 2.6 Disinfectant efficacy testing on CDC reactor cultivated *S. aureus* biofilms

A modification of ASTM method E2871 (single tube method) was used to gain a better knowledge of derivative efficacy. Log reduction in viable biofilm cells exposed to 90 μM (4 × IC_50_) of **A3**, 100 μM CHX and 400 μM of penicillin G was measured for 1 h, 2 h and 3 h [33]. Briefly, coupons containing biofilms of *S. aureus* AH2547 were removed from the CDC reactor, rinsed and then transferred to 50 ml conical tubes with tweezers. Subsequently, 4 ml of TSB solution or compound containing TSB solutions were carefully added to the tubes, and the tubes were incubated at 37 °C under static conditions. At each specific time point, 36 ml D/E broth for compound neutralization purposes were added and biofilms were disaggregated by sonication and vortexing according to ASTM E2871. The diluted samples were drop plated on TSA plates, incubated overnight at 37 °C and enumerated. D/E broth was validated to neutralize **A3** to concentrations up to 200 μM according to the procedure in the standard test method for evaluation of inactivators of antimicrobial agents (ASTM E1054) (data not shown).

### 2.7 Treatment flow cell assay with detection of membrane integrity and biomass removal by confocal microscopy

A dual dye leakage indicator system was established by using the FilmTracer™ Calcein red-orange biofilm stain (Thermo Fisher Scientific, Waltham, MA, USA) and bacterial expression of GFP. Fading due to diffusion of the small calcein dye (Mw 789.55 Da) from the biofilm was intended to indicate small pore formation which would to a lower extent affect the leakage of the intracellular, large GFP (238 amino acids, 27 kDa). Fading of both calcein-red-orange and GFP, would thus indicate formation of large pores or membrane collapse. Biofilms were stained with the FilmTracer™ Calcein red-orange dye according to the manufacturer's instructions. After rinsing the coupons for removing unbound stain, the coupons were transferred to the treatment flow cell (model FC310; Bio Surface Technologies, Bozeman, MT, USA) and video microscopy experiments were conducted as described previously [39]. In brief, untreated controls were treated with full strength TSB supplemented with DMSO as vehicle control. Images were acquired of the bright field, GFP, and RFP channel using a Leica SP5 confocal laser-scanning microscope. The z-stack step size was set to 10 μm.

### 2.8 Software and statistical analysis

SigmaPlot 14.5 (Systat Inc., Chicago, IL, USA), was used for plotting of graphs and statistical analysis (Student's t-test). Included asterisks in figures indicate significant differences with *p ≤ 0.05, **p ≤ 0.01, ***p ≤ 0.001. A p-value of < 0.05 was considered statistically significant.

Overlay images of treatment flow cell results were created with Adobe Photoshop CS6 (Adobe Systems Inc., San Jose, CA, USA) whereas movie generation was carried out with IMARIS® (Bitplane AG, Zurich, Switzerland).

## 3 Results

### 3.1 A1, A2 and A3 affect *S. aureus* biofilm viability within 1 h

**Fig. 2:**
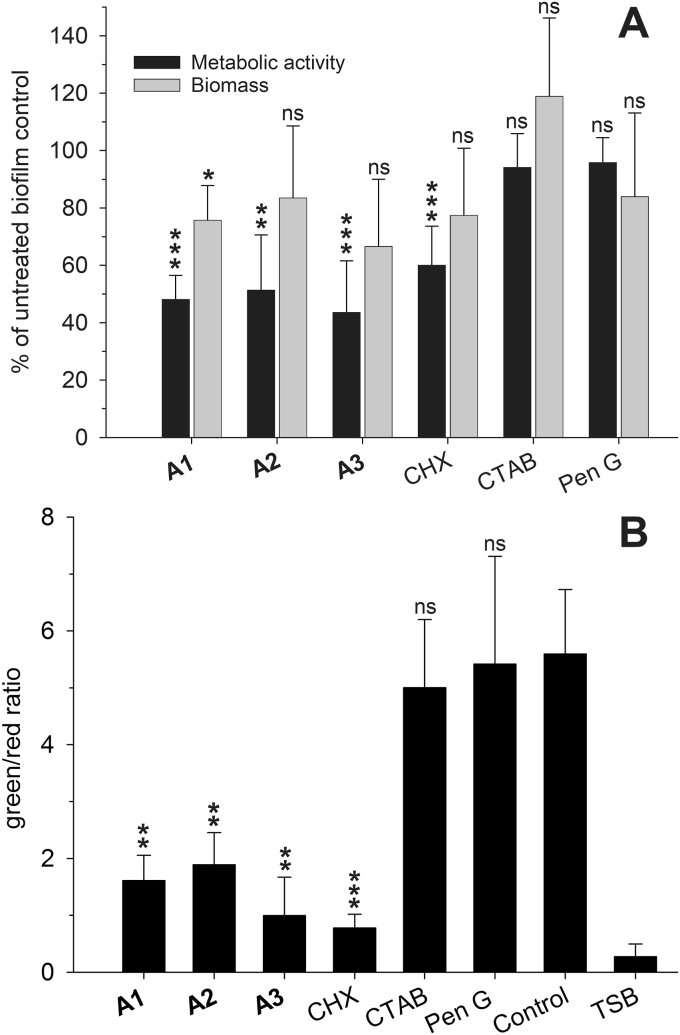
**Metabolic activity, biomass and membrane integrity assays with *S. aureus* ATCC 25329.** (A) Resazurin based metabolic activity assessment and crystal violet based biomass staining. (B) Detection of the green/red ratio in TSB containing wells, untreated control biofilms and treated biofilms bacteria for assessment of membrane integrity assessment based on the green SYTO 9 and red PI dye. (Results shown under (A) and (B) display the mean with standard deviation of at least three independent experiments).

The initial experiments investigated the immediate impact of different treatments on *S. aureus* (ATCC 25329) biofilms. The α,α-disubstituted β-amino amides **A1** - **A3** reduced biofilm viability to approximately 50% after 1 h of treatment at concentration of 45–50 μM (Fig. 2 A). We observed comparable effects after treatment with 50 μM of the disinfectant CHX. In contrast, treatment with 50 μM of the quaternary ammonium compound CTAB or 400 μM of Pen G did not reduce biofilm viability even though these compounds inhibited planktonic *S. aureus* ATCC25329 at 2.5 μM and 0.12 μM (TAB S1). Substantial biofilm removal was not observed for any of the treatments, however, derivative **A3** yielded approximately 30% removal. CHX seemed to act similarly to the α,α-disubstituted β-amino amides and appeared as a suitable control for further studies. The presence of human serum albumin did not significantly lower the potency of the derivatives **A1** - **A3** or CHX (Fig. S1). However, CTAB and Pen G suffered loss of activity under the presence of the plasma protein (Fig. S1).

### 3.2 A1, A2 and A3 cause membrane permeabilization in *S. aureus* biofilms

The SYTO 9/propidium iodide assay is useful for assessment of bacterial cell membrane integrity. Intact bacteria are stained green with the membrane permeable dye SYTO 9. Bacteria with compromised membranes are additionally susceptible to staining with the red fluorescent dye PI, which is otherwise excluded from viable cells due to its positive charge. Derivatives **A1** - **A3** as well as CHX showed increased PI staining, i.e., absence of any barrier function of the bacterial membrane within 1 h of treatment with 45–50 μM, compared to 50 μM CTAB, 400 μM Pen G or untreated controls (Fig. 2B).

**Fig. 3:**
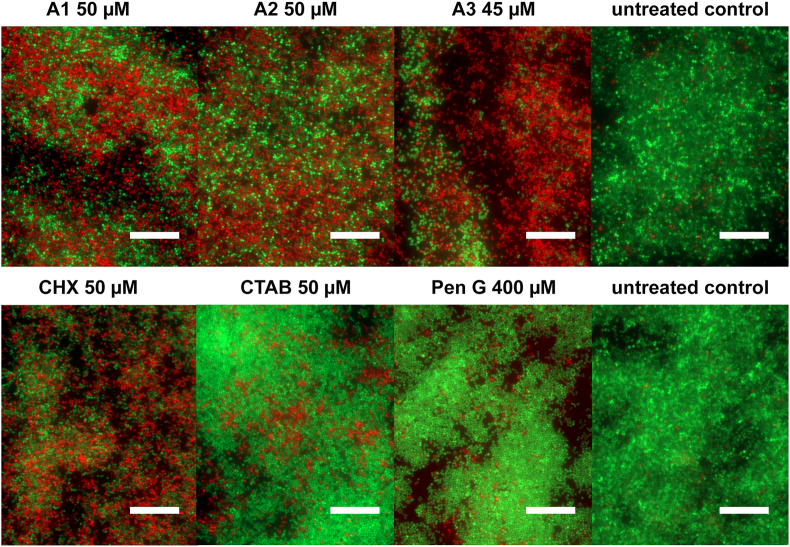
Fluorescence microscopy of *S. aureus* ATCC 25329. Biofilms were treated 1 h with derivatives and controls and stained with the green SYTO 9 and red PI dye staining kit. Green represents intact cells whereas red indicates bacteria with compromised membrane integrity. Scale bars represent 200 μm. (For interpretation of the references to colour in this figure legend, the reader is referred to the Web version of this article.)

The SYTO 9/propidium iodide staining is additionally suitable for fluorescence microscopy studies. Imaging of treated and subsequently stained biofilms (Fig. 3) showed similar staining patterns as observed in the quantitative well-plate based fluorescence measurements (Fig. 2B). The most pronounced PI staining was noted when the biofilm was treated with **A3** and CHX, and to a slightly lower extent for the **A1** and **A2** treated biofilms. In contrast, only a few bacteria were PI positive in the CTAB and Pen G treated biofilms. Minimal PI staining was detected in the untreated control biofilms.

### 3.3 α,α-disubstituted β-amino amides cause ATP efflux from *S. aureus* biofilms

**Fig. 4:**
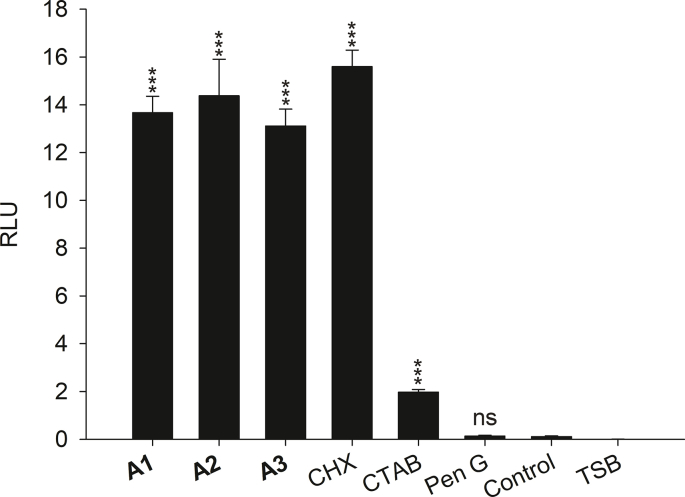
**ATP release assays with *S. aureus* ATCC 25329.** ATP release as measure of membrane integrity of biofilm bacteria by ATP - luciferin/luciferase-based luminescence assay. (Results display the mean with standard deviation of at least three independent experiments).

The ATP dependent luciferin-luciferase reaction was utilized to investigate membrane damage and ATP leakage upon incubation with the test compounds. The assay showed that α,α-disubstituted β-amino amides as well as CHX caused a pronounced release of ATP and supported our findings regarding disrupted cell integrity of derivative or CHX treated biofilm bacteria (Fig. 4). The surface-active compound CTAB showed ATP release to a much lower extent compared to the derivatives and CHX. Released ATP after Pen G treatment was negligible and comparably low as observed for untreated controls.

### 3.4 Log reduction on CDC reactor cultivated *S. aureus* AH2547 biofilms by A3, CHX and pen G

**Fig. 5:**
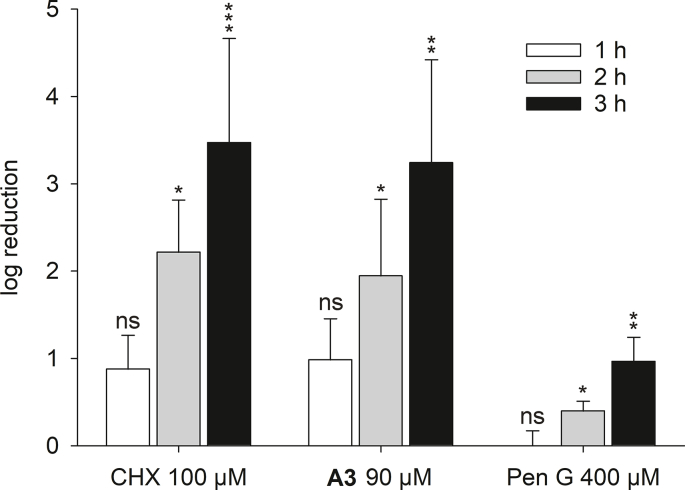
Determination of log-reduction in viable cells of *S. aureus* AH2547 biofilms by single tube method. Coupons were sampled from the CDC biofilm reactor and subsequently exposed to treatments for 1 h, 2 h and 3 h. After enumeration, the log (colony counts) of treated biofilms were subtracted from log (control counts) resulting in log reduction. At least two coupons for each treatment were used and a minimum of three independent experiments performed (results display the mean with standard deviation).

Preliminary experiments in 96-well plates indicated that **A3** was the most potent of our derivatives against planktonic *S. aureus* AH2547 and able to prevent biofilm formation (Fig. S2). Additionally, susceptibility of preformed *S. aureus* AH2547 biofilm was assessed in 96-well plates and showed it was necessary to increase the concentration of the derivatives to 4xIC_50_ to achieve a biofilm reduction of 40% or higher. A concentration of 90 μM of **A3** for 3 h reduced preformed biofilms to the same extent as was achieved after a 3 h of treatment with CHX. CHX with a concentration of 100 μM was used as control based on doubling the concentration of **A3**. These assay parameters were selected for the single tube method experiments. The single tube method is a validated standard test method well suited for determination of anti-biofilm efficacy testing [40]. Derivative **A3** at 90 μM and CHX at 100 μM killed biofilm cells at a rate of approximately 1 log unit per hour (Fig. 5). After 3 h, both compounds showed a 3.5 log reduction (99.97% reduction of biofilm) of viable cell counts whereas Pen G showed a reduction of only 1 log unit.

### 3.5 A3 causes membrane permeabilization and biomass removal of *S. aureus* biofilms under flow conditions

**Fig. 6:**
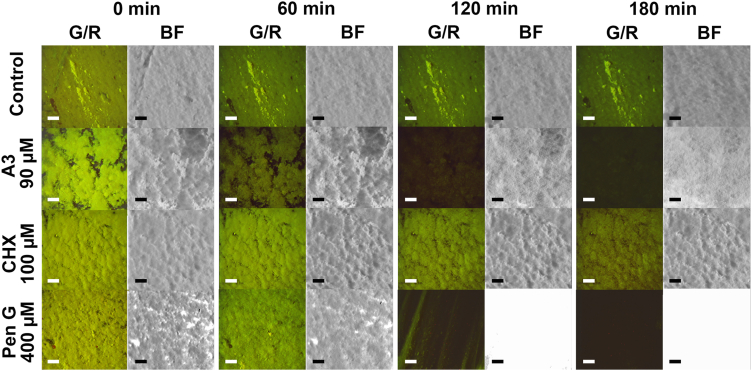
Treatment flow cell/CLSM video microscopy experiments of *S. aureus* AH2547 biofilms. GFP expressing and calcein red-orange stained biofilms as overlay images with green/red (G/R) fluorescence. Bright field images reveal the presence of biofilm (dark) independent of fluorescence and facilitate interpretation of the impact on membrane integrity and biofilm removal during a period of 3 h. Dark G/R images indicate loss of fluorescence and/or non-fluorescent biofilm structures whereas white bright field images indicate absence of biofilm. Scale bar represents 100 μm. (For interpretation of the references to colour in this figure legend, the reader is referred to the Web version of this article.)

The effect of **A3** and CHX under flow conditions was assessed using the treatment flow cell. The treatment flow cell is a useful tool to investigate removal properties of biofilm treatments and is engineered to fit coupons sampled from a CDC biofilm reactor. The assessment of biofilm killing efficacy and removal events using coupons collected from a single CDC biofilm reactor experiment can be performed, as described previously [39]. No morphology changes were noted in the bright-field or fluorescence images when the biofilm was treated with full-strength TSB (Fig. 6 and Fig. S4). In contrast, treatment with 90 μM **A3** resulted in a decrease of fluorescence intensity after 60 min and fluorescence loss was additionally intensified by removal of biofilm over the remaining 120 min. The biofilm bacteria membranes seemed to disintegrate after 60 min since both GFP and calcein red-orange faded simultaneously before dispersal of the biofilm. CHX (100 μM) showed a similar effect on AH2547 biofilms, however, fluorescence loss was less pronounced compared to the **A3** treated biofilms. In addition, the biofilm appeared to develop a rougher topography in the bright field images, and did not disperse as promoted by **A3**. Treatment with Pen G (400 μM) started with erosion and resulted in complete dispersal of the biofilm indicated by removal of visible structures in both the fluorescent and bright field images.

## 4 Discussion

The mode-of–action studies showed that α,α-disubstituted β-amino amides are capable of efficiently killing and removing biofilms. Easily cultivable biofilms in 96-well plates and use of known assay parameters from our previous antibiofilm screening studies facilitated our approach [31]. The resazurin based metabolic activity assays showed that the most potent derivatives **A1**, **A2** and **A3** were able to decrease biofilm viability at 45–50 μM within 1 h, similar to the cationic control disinfectant CHX (Fig. 2A). Surprisingly, CTAB did not affect biofilm viability to the same extent as CHX even though we measured similar susceptibility of planktonic *S. aureus* (TAB S1). Similar to values reported by Manner et al., Pen G at 400 μM was not active against the biofilms [38]. In microtiter plate assays, little biofilm removal was observed for any treatment, which suggested that biofilm removal was time dependent/flow dependent for these compounds or a downstream event after killing of biofilm bacteria. To our surprise, the presence of 300 μM human serum albumin (HSA), which is present in wound exudates [41], did not inhibit the potency of the derivatives. Tetra- and hexapeptides which contained α,α-disubstituted β-amino amides as building blocks showed decreased activity against lymphoma cells, when co-incubated with HSA for 6 h [42]. Binding of lipophilic groups to HSA was located on drug site II. Similar results were obtained for other small synthetic mimics of antimicrobial peptides when HSA (550 μM) was present during MIC assays with various bacterial strains [43]. Of note, Sivertsen et al. suggested that lipophilic side chains larger than a benzyl group may reduce binding to HSA [44]. That α,α-disubstituted β-amino amides were not affected by HSA may be attributed to their bulky lipophilic groups. In addition, the derivatives were used in high concentrations over 24 h against adherent biofilms compared to shorter treatment of cells/bacteria in suspension [42,43]. Due to their fast mechanism of action, release of biofilm constituents may have interacted with HSA during the 24 h incubation time and thus reduced HSA binding properties. In contrast, potency of CTAB and Pen G decreased which may be due to the reported high affinity of HSA to bind acidic drug molecules and molecules with long aliphatic chains [45,46].

The quantitative and qualitative green SYTO 9 and red PI dye uptake analyses (Fig. 2, Fig. 3) confirmed the viability data. During these experiments approximately 50% of *S. aureus* biofilms showed signs of membrane damage within 1 h of treatment with **A1**, **A2** and **A3**. Similar results were obtained with CHX even though incorporation of the hydrophobic moiety in microbial membranes plays only a minor role in the proposed mode-of-action for CHX [32]. Treatment with the cationic derivatives **A1** - **A3** resulted in membrane damaging effects as seen with CHX, whereas CTAB or Pen G did not show any membrane damaging properties during the incubation period. Thus, CTAB appeared to be unable to perturb microbial membranes under the experimental conditions, which also explains its low impact on biofilm viability. It has been reported that staining with SYTO 9 and PI can result in mixed states, which make differentiation between live and dead bacteria challenging [47]. Therefore, we additionally performed a membrane disintegration study based on the leakage of intracellular ATP. The ATP assay confirmed the SYTO 9 and PI staining experiments. Treatment with derivatives **A1** - **A3** as well as CHX resulted in a considerable leakage of ATP. Similar observations of ATP leakage due to pore formation were reported for small, dehydroabietic acid derived compounds and the bacteriocin Nisin A [38,48]. We observed only a minor ATP leakage after CTAB treatment and no effect after incubation with Pen G.

Biofilms of GFP expressing *S. aureus* strain AH2547 grown in a CDC biofilm reactor allowed us to challenge the α,α-disubstituted β-amino amides in a different assay system. We adapted the standardized ASTM method 2562 [36] to grow CDC reactor biofilms under higher shear forces and for a longer time compared to the 96-well plate setup. Susceptibility of planktonic *S. aureus* AH2547 against our derivatives was in the same range as previously described for *S. aureus* ATCC25923 [31]. However, *S. aureus* AH2547 biofilms seemed more tolerant to **A3** treatment, similar to our previous observations with the *S. aureus* Newman strain [31]. Treatment of harvested *S. aureus* AH2547 biofilms with 90 μM **A3** or 100 μM CHX resulted still in equally high log reductions and kill rates of over 99.99% (Fig. 5). These findings in combination with our results from our 96-well plate assays suggested a similar mode-of-action of **A3** and CHX. These two compounds have an equal number of covalent bonds between the two positively charged moieties. The two (p-chlorophenyl)guanide units in CHX are linked by a six carbon hexamethylene bridge, which are important for its activity by bridging two neighboring phospholipid head groups [32]. This results in inhibition of metabolic functions of the membrane and can ultimately lead to structural integrity loss, which may also be the case for our derivatives. However, the treatment flow cell experiments revealed a notable difference between CHX and **A3** regarding their biofilm removal properties (Fig. 6). We found indications that **A3** was able to additionally remove biofilm. *In silico* simulations have shown that **A2** and **A3** only partly incorporate the hydrophobic moiety into bacterial membranes (“can-can” pose which also correlates with greater anti-biofilm activity) in contrast to the proposed mode-of-action of CHX [32,49]. Koivuniemi et al. suggested that the insertion of only one hydrophobic side chain may lead to local aggregation of the compounds driven by the hydrophobic effect which results in a collective behavior of these compounds that disrupts the bacterial membrane [49]. Additionally, an exposed hydrophobic arm may impact the protective peptidoglycan macronet outside of the bacterial membrane. The observed effects of membrane disintegration as well as decreased biofilm cohesion in the treatment-flow-cell experiments would fit with the proposed behavior for **A2** and **A3**. However, further studies are needed to pinpoint localization and interaction of α,α-disubstituted β-amino amides in biofilms. Simultaneous killing and removal of biofilm is a favorable outcome for anti-biofilm treatments because of maintenance of tissue/medical device functionality and limiting the chance of biofilm re-establishment on a preconditioned surface. Therefore, biofilm removal is an equally important biofilm controlling strategy in addition to killing the biofilm as highlighted by Gloag et al. [50]. For instance, the non-biocidal peptide A has been shown to effectively disperse and prevent biofilms also after grating to surfaces [11,51]. Various antimicrobial treatments such as glutaraldehyde, the antimicrobial peptide Nisin, or quaternary ammonium compounds are able to kill biofilm bacteria, however, they all lack the capability to remove the biofilm [52]. Even though the bactericidal impact of CHX can contribute to biofilm erosion, a considerable amount of biomass remained in comparison to treatment with **A3** or Pen G. Crosslinking of anionic matrix components has been suggested for CHX's lack of removal properties, and a decrease of biofilm deformability after CHX treatment has been observed [53,54]. Even though **A3** is di-cationic, it did not share the inability of reported cationic compounds of removing biofilm biomass.

Our results show that α,α-disubstituted β-amino amides affect *S. aureus* biofilms as fast as the antiseptic CHX. Biofilm treatment leads to membrane permeabilization and application of these derivatives under flow conditions causes biofilm removal. These properties make α,α-disubstituted β-amino amides attractive candidates for the development of new anti-biofilm control strategies.

## CRediT authorship contribution statement

**Dominik Ausbacher:** Conceptualization, Methodology, Investigation, Formal analysis, Writing – original draft, Writing – review & editing, Funding acquisition, Project administration. **Lindsey A. Miller:** Methodology, Investigation, Writing – review & editing. **Darla M. Goeres:** Conceptualization, Methodology, Resources, Supervision, Validation, Writing – review & editing. **Philip S. Stewart:** Conceptualization, Methodology, Resources, Supervision, Writing – review & editing. **Morten B. Strøm:** Conceptualization, Resources, Supervision, Writing – review & editing, Funding acquisition, Project administration. **Adyary Fallarero:** Conceptualization, Methodology, Resources, Supervision, Validation, Writing – review & editing.

## Declaration of competing interest

The authors declare the following financial interests/personal relationships which may be considered as potential competing interests. Given her roles as an Editor, D.M. Goeres had no involvement in the peer review of this article and had no access to information regarding its peer review. Full responsibility for the editorial process for this article was delegated to a different editor. A. Fallarero is currently employed by Thermo Fisher Scientific, but this work has no association with her current employment relationship. The other authors declare that they have no known competing financial interests or personal relationships that could have appeared to influence the work reported in this paper.

## Data Availability

Data will be made available on request.

## Appendix A Supplementary data

Supplementary information Supplementary information

Fig. S4 (A) Exposure to full-strength TSB for 3h and 3 min. Fig. S4 (A)

Fig. S4 (B) Exposure to full strength TSB (3 min) and subsequently to A3 (90 µM) for 3h. Fig. S4 (B)

Fig. S4 (C) Exposure to full strength TSB (3 min) and subsequently to CHX (100 µM) for 3h. Fig. S4 (C)

Fig. S4 (D) Exposure to full strength TSB (3 min) and subsequently to Pen G (400 µM) for 3h. "Fig. S4 (D)
