# Supplementary material for: α,α-disubstituted β-amino amides eliminate Staphylococcus aureus biofilms by membrane disruption and biomass removal
Source: Biofilm. 2023 Aug 25;6:100151. doi: 10.1016/j.bioflm.2023.100151 (PMC10474319; doi:10.1016/j.bioflm.2023.100151)
Supplement: Supplementary information [file mmc1.docx]

**Supplementary information**

**Table S1. MIC data of chlorhexidine (CHX), cetyltrimethylammonium bromide (CTAB) and penicillin G (Pen G) against planktonic *Staphylococcus aureus* ATCC 25329**

|  | **MIC of control compounds [µM]** | | | |  |
| --- | --- | --- | --- | --- | --- |
| **CHX** | | **CTAB** | | **Pen G** | |
| **2.25** | | | **2.50** | **0.12** | |

**
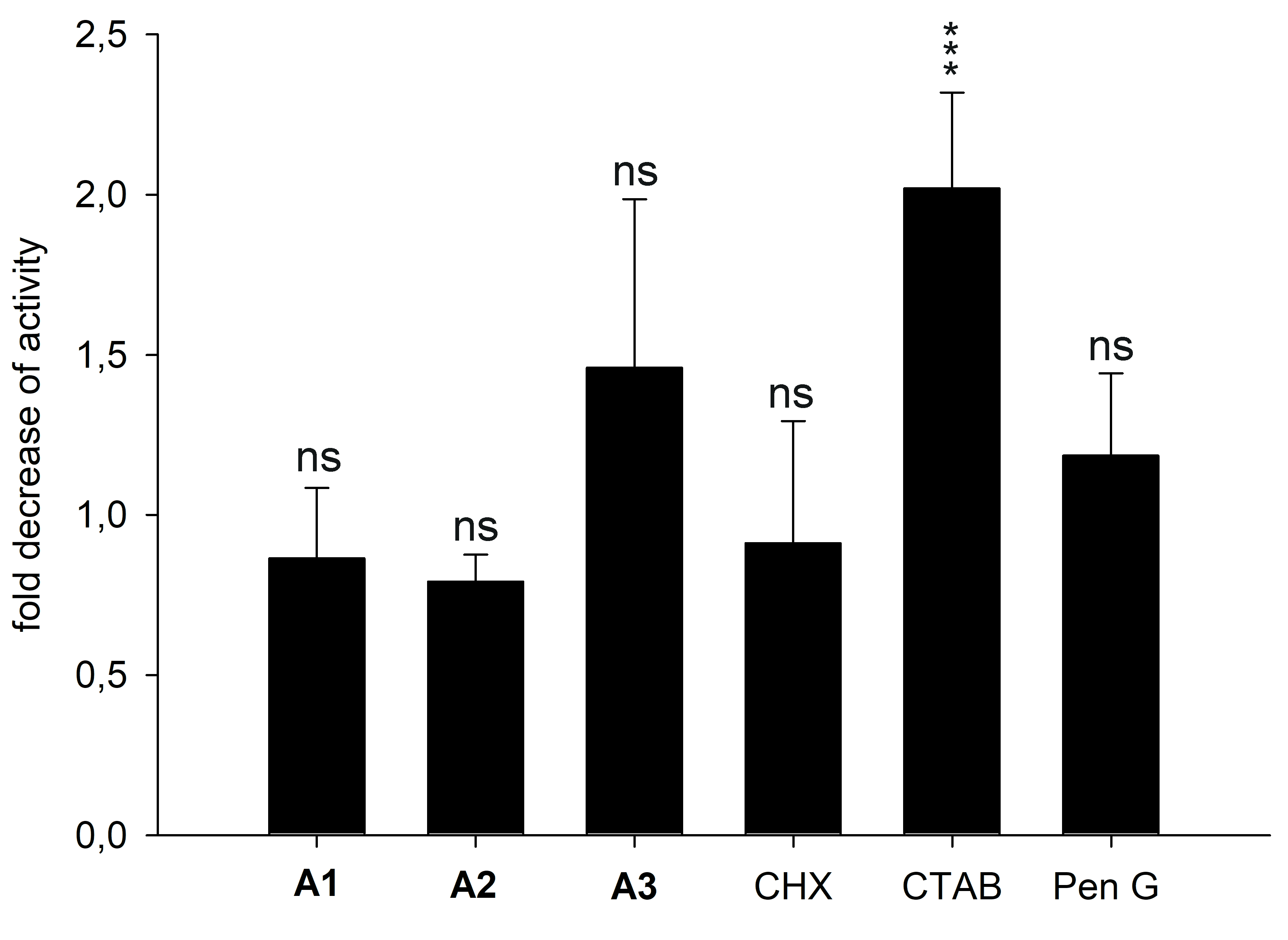
**

**FIG S1. Impact of human serum albumin on anti-biofilm potency by resazurin based metabolic activity assessment.** *S. aureus* ATCC 25923 biofilms were formed in 96-well plates for 18 h. The biofilms were concurrently treated with the α,α-disubstituted β-amino amides and control substances in the absence and presence of 300 µM HSA (>99%, Sigma-Aldrich, Schnelldorf, Germany), e.g. representing HSA levels in wound exudate [1], for 24 h. After applied resazurin staining, treatment efficacies were compared. (Results display the mean with standard deviation of three independent experiments).

**

**

**FIG S2. Susceptibility of planktonic *S. aureus* AH2547.** MIC data of **A1** - **A3** and CHX and biofilm prevention after replacement of the planktonic phase with pure TSB. (Bars represent the mean of three independent experiments and error bars indicate the 95% confidence interval).

**

**

**FIG S3. Susceptibility assessment of *S. aureus* AH2547 biofilms cultivated in 96-well plates over 18 h.** The pre-formed biofilms were treated with chlorhexidine and α,α-disubstituted β-amino amides for 1 - 3 h. After exchange of the planktonic phase, GFP fluorescence of the remaining biofilm was detected, and compared with untreated controls (results display the mean ± SD of three independent experiments.

**Figure S4 (online version of the article). Representative time-lapse video of treatment-flow-cell experiments displaying the GFP, RFP and bright field channels.** (A) Exposure to full-strength TSB for 3h and 3 min. (B) Exposure to full strength TSB (3 min) and subsequently to **A3** (90 µM) for 3h. (C) Exposure to full strength TSB (3 min) and subsequently to CHX (100 µM) for 3h. (D) Exposure to full strength TSB (3 min) and subsequently to Pen G (400 µM) for 3h.
